# Returning to Work After Stroke: Perspectives of Employer Stakeholders, a Qualitative Study

**DOI:** 10.1007/s10926-012-9401-1

**Published:** 2012-12-05

**Authors:** Carol Coole, Kathryn Radford, Mary Grant, Jane Terry

**Affiliations:** 1 Faculty of Medicine and Health Sciences, University of Nottingham, A Floor, South Block Link, Queens Medical Centre, Nottingham, NG7 2HA UK; 2 Division of Rehabilitation and Ageing, University of Nottingham, B Floor, Medical School, Queens Medical Centre, Nottingham, NG7 2UH UK; 3 Community Stroke Team, Newbrook House, 385 Alfreton Road, Nottingham, NG7 5LR UK

**Keywords:** Stroke, Qualitative research, Employers, Work, Vocational rehabilitation, Return to work

## Abstract

*Purpose* More than 40 % of working age adults with stroke fail to return to work. The work context is a key factor in return to work, but little is known about the experiences of employers in supporting employees with stroke. The aim of this study was to explore return to work after stroke from the employer perspective, to identify key features associated with success and to seek participants’ views regarding the role of healthcare in return to work. *Methods* Data was gathered through 18 semi-structured interviews with employer stakeholders and included small business owners, line managers, human resources and occupational health staff. Data was analysed thematically. *Results* The main themes identified were: the impact of stroke on the employer, characteristics of the employee, communication, knowledge and information, experience of other stakeholders, integrating healthcare in return to work. *Conclusion* Employers face complex emotional and practical issues when helping an employee return to work after stroke, for which many lack knowledge and experience. The range and quality of support networks that they access is variable and advice and support from clinicians is welcomed. Further research is necessary to investigate how such support could be funded and integrated within existing service provision.

## Background

Of the 150,000 people who suffer a stroke each year in the UK approximately a quarter are of working age [1]. The societal cost in terms of health and social care, informal care giving and lost productivity are estimated at £9 billion a year [2].

Returning to work (RTW) is a primary rehabilitation goal yet reported success varies widely [1, 3]. In a systematic review examining the social consequences of stroke in working aged adults, Daniel et al. [1] found that of the 8,810 stroke survivors working before stroke, only a mean 44 % (range 0–100 %) returned to work. Similar figures are reported in national prevalence surveys in Japan and Sweden, with higher proportions among younger stroke survivors who were working at onset [4, 5]. It is not only returning to work that presents a problem; ensuring people remain in work is also difficult. Stroke survivors may return prematurely and leave once the true impact of the stroke on their job is realized [6].

Vocational rehabilitation (VR) has been defined as whatever helps someone with a health problem return to, and remain in, work [7]. It involves helping people find work, helping those who are in work but having difficulty and supporting career progression in spite of illness or disability [8, 9]. For the stroke survivor in the UK VR is concerned with seeking ways to develop a ‘match’ between the abilities and limitations of the stroke and the demands of the job and the work environment, and considers the interaction of physical, emotional, cognitive, environmental, organisational and social factors on work ability. It involves assessing people’s functional capacity for work, job evaluation, safety assessment, liaison with employers about the need for equipment or adaptations to the job and or work environment and educating the employer, patient and his/her family about the effects of stroke and their legal responsibilities. It may also involve re-training and negotiating work trials and brokering placements for those unable to resume an existing job [10]. UK Government policy [11] and clinical guidelines [10–13] stipulate that VR support should be provided and keeping people with long term conditions in work is a recognized National Health Service (NHS) health outcome [14]. Despite this, not all stroke survivors in the UK have access to this level of support. Health based services supporting people with stroke in returning to work are rare in the UK and meet less than 10 % of the estimated need [9, 15, 16]. Stroke rehabilitation in the NHS does not typically extend to vocational rehabilitation. It focuses on getting people home from hospital and their physical recovery. Pressures on reducing the length of hospital stay mean that the needs of people with milder strokes or hidden deficits (such as impaired insight, executive dysfunction, anxiety and fatigue) are frequently overlooked [17]. Since the focus of many existing Government employment services (Department for Work and Pensions—DWP) initiatives is on re-training or returning people to work following long periods of sickness absence, these only activate at the point when a person transfers from sickness onto incapacity benefits (at around 6 months after illness/injury onset). This means that many stroke survivors are not supported at a time when they need help and may lose their jobs or relinquish work because of a lack of timely VR intervention [6].

Black and Frost [18] call for early health based interventions to prevent job loss (by keeping the door to an existing employer open) and recognise that supporting those who have the capacity to work is an important role for health care professionals [19]. However, since VR is also concerned with addressing employers’ expectations, healthcare cannot address the vocational needs of stroke survivors in isolation. The work context is an important determinant of return to work success [4, 5, 20–22] yet little research has focused on the employer perspective in vocational rehabilitation generally [23, 24] or specifically following stroke [25].

In the UK a number of different agencies could potentially support employers in returning stroke survivors to work. Occupational health services employ physicians and nurses whose role it is to advise the employee and their line manager on their fitness to work. However, these only tend to be offered in large public sector organisations and larger private companies. Additional VR support may be available through insurance providers in the private sector, advisors and web based information in the voluntary sector e.g. The Stroke Association, and the UK government’s Department for Work and Pensions’ employment advisers and employment services. However, many small businesses don’t have access to occupational health services and as stroke does not result from accidental injury, VR providers in the private sector tend not to be involved. Voluntary sector services, like health are haphazard, depend on local commissioning arrangements and may lack VR expertise. As support from government employment services is only activated later and stroke is regarded as a legitimate disability and not a priority (in terms of conditions constituting the greatest proportion of state benefit claimants who are out of work), then stroke survivors are rarely supported and where they are this may be by people without any stroke specific knowledge [17]. Larger organisations may have Human Resources (HR) departments within organisations who are responsible for recruiting, training, retaining and maintaining an effective and efficient workforce including sick or disabled employees, and can support ‘line managers’ or supervisors to manage an employee’s return to work.

In the UK, the National Institute for Health Research (NIHR) has commissioned collaborative partnerships, known as CLAHRCs (Collaborative Leadership in Applied Health Research and Care) between academic partners, healthcare providers, patients and commissioners, to tackle the known difficulty of translating research evidence into everyday healthcare practice. Stroke Rehabilitation is one research theme in the Nottinghamshire/Derbyshire/Lincolnshire CLAHRC partnership. The theme includes a feasibility randomised controlled trial of an occupational therapist-led stroke-specific return-to-work intervention and studies investigating the barriers and enablers to the development of a healthcare-based post-stroke vocational rehabilitation service, including this one. The aim of this study was to explore the experiences and perceptions of employer stakeholders in supporting RTW of workers following stroke, to identify key features associated with successful RTW and to seek stakeholders’ views regarding a therapy-led VR RTW service.

## Methodology

### Design

Qualitative methodology was used and data collected through semi-structured interview. The aim was to conduct a minimum of ten interviews with employer stakeholders. Potential recruitment mechanisms/sources were identified by the research team in consultation with the project steering group. Participants were recruited through a variety of means: personal contact with the study steering group and expert panel (who included stroke service users and providers), an on-line support group for stroke survivors, the website of a small businesses federation and by personal approach to occupational health providers and human resources departments of large organisations. Participants were offered a choice of face-to-face or telephone interviews at a time and location convenient to them. Purposeful sampling was initially employed to recruit stakeholders with experience of supporting an employee return to work following a stroke; however, due to initial recruitment difficulties convenience sampling was used. Interviews were digitally recorded and transcribed verbatim by an approved transcription service and checked by the researcher who conducted the interview. Written consent was obtained. A list of topic areas using open questions and prompts was developed through a review of the literature and discussion with the research team. Topics included the participant’s experience and perceptions of employing stroke survivors, any support the participant may or may not have received from both within and external to the workplace and their views and recommendations as to future support mechanisms and how these might be funded. Additions to the guide were made in response to new topics arising as the interviews progressed, and the guide was adjusted for use with participants who did, and did not, have direct experience of supporting an employee returning to work after stroke.

Ethical approval was granted by the Leicester Research Ethics Committee.

### Data Analysis

The data was analysed thematically [26]. A qualitative software package, NVivo9, (QSR International Pty Ltd), was used to manage the data. The research team included an occupational therapist experienced in delivering health-based vocational rehabilitation [CC] three occupational therapists experienced in supporting stroke survivors in a return to work [KR, MG, JT] a social scientist [KS] and research assistant [ES]. Three members of the team had experience in analysis of interview data [CC, KR, KS]. Initial coding was conducted by the main interviewer [CC] following constant comparison of the transcripts as the data was collected, and independently by a second researcher [KR]. In order to increase validity and reliability of the analysis, a selection of transcripts and the suggested codes were then discussed and revised with the wider research team [CC, KR, MG, JT, ES, KS]. The team included those members who had conducted two of the earlier interviews. Following these meetings, codes were refined further and agreed with the second researcher [KR]. Initial themes were the identified by the main researcher [CC], discussed with the wider research team, and finally agreed with the second researcher [KR].

## Results

A total of 18 participants were recruited. Sixteen of the interviews were conducted by one researcher [CC] between November 2011 and February 2012. Data from two previous face-to-face interviews, conducted by other members of the research team in January and August 2011 [KS, MG, JT], and which had not been analysed, were added. The mean duration of each interview was 53 min (range 27–92 min). Details of the stakeholder groups represented by participants can be seen in Table 1. All were located in an urban environment. Two of the participants had themselves had a stroke, one of whom had returned to work. Both worked in managerial positions. Fourteen of the participants had recent experience of supporting an employee return to work after stroke, twelve of these within the last 2 years.

**Table 1:** Details of the participants

Stakeholder group	N	Type of industry
Human resources		
Public sector (large±)	2	Service
Voluntary sector (large)	1	Service
Occupational health advisers		
Physician—private sector, in-house (large)	1	Manufacturing
Nurse—public sector, in-house (large)	1	Service
Nurse—private sector, in-house (large)	1	Manufacturing
Nurse—nationwide private provider	1	Various
Owners of small businesses (micro)	3	Service
Managers		
Managing Director—private sector (medium)	1	Engineering
Manager—voluntary sector (micro)	1	Service
Line manager/supervisor—public sector (large)	2	Service
Line manager/supervisor—private sector (medium)	2	Engineering
Line manager/supervisor—private sector (small)	1	Service
Disability adviser (voluntary sector)	1	Service

±size of workforce: large: >250, medium: >50–250, small: 10–50, micro: <10

## Interview Findings

The themes and sub-themes identified through analysis of the interview data can be seen in Table 2. These are described in detail with quotations to illustrate the themes.

**Table 2:** Themes and sub-themes identified through analysis of the interview data

The impact of stroke on the employer
The emotional response
Employers’ concerns
Characteristics of employees returning to work after stroke
Personal characteristics
How employers perceive the effect of stroke on the individual employee
Motivational factors
Awareness/acceptance of limitations
Communication
Asking for help
Consent and confidentiality
Communication between healthcare and the workplace
Knowledge and information
Employers’ understanding of stroke
How employers gain knowledge about stroke
Awareness of disability management
Awareness of existing support services
Experiences and perceptions of other stakeholders
GPs/physicians
Occupational health (OH)
Management (Human Resources (HR), line manager/supervisor)
Integrating healthcare in return to work
Content and delivery
Who might pay?

## The Impact of Stroke on the Employer

### The Emotional Response

Participants described a range of emotional responses to the experience of managing an employee with stroke, in relation to both the condition itself and the return to work process. If this was a current employee, participants described a sense of shock and disbelief in response to an event that was sudden and unexpected. This response could affect the whole team, with more significant impact in small workforces.

It was devastating at the time, just the things that happened….it was horrendous. (Participant 11, business owner)

As in the above case, the individual may have been a long-term employee and a close colleague of the manager. Not only did managers have to cope with their own and their teams’ reactions to the individual with stroke, but also their reflections of their own mortality. In addition there was the impact of reduced staffing, the short or longer-term loss of that individual’s experience and knowledge, together with the demands of supporting the individual during their recovery (e.g.maintaining contact and visiting them while in hospital or at home) and during their return to work. Where employees had not returned to work or where the return to work had not been sustained, as in the following cases, managers might experience sadness, anxiety, guilt or anger:

we both got upset and had a hug because we’ve known each other for so long and she’s a really good worker, she loved her job….(Participant 5, line manager)

You never see something like that coming…….it’s a period that I’ll never forget…………but should I have gone and found out myself [about how to help the employee stay at work] that’s what I question? (Participant 11, business owner)

However, in contrast, supporting an employee could also be a positive, rewarding and enjoyable experience.

Well, for me personally, it’s been, this sounds all wrong, but it’s been an enjoyable experience because it’s been really interesting, trying to understand what happened to [the employee] and how it affects him and how it affects his response time. (Participant 7, line manager)

### Employers’ Concerns

Employers were concerned about managing an employee with stroke for a number of reasons. These included the uncertainty of how stroke might affect the individual’s capacity to work, concerns about how a return to work might make their health condition worse or cause another stroke, which they perceived might happen at work.

Unfortunately we have had somebody die on the premises of a heart attack and so we knew the effect that that could have, you know, emotionally on people around and took a long time for people to forget - and the consequences of him having a stroke at work and the people around him and how that affects them and, you know, that was just a huge problem. (Participant 8, managing director)

Concerns could be experienced by employers and colleagues before return to work and also subsequently:

She (the employee’s colleague) would have her concerns just the way that (the employee) would look some days or behave. Memory loss things and just not looking well. And there was one particular day where a client did have to go downstairs and get (another staff member) because (the employee) came over all funny.

(Participant 11, business owner)

In neither of these cases had the employee successfully sustained their return to work.

There was concern about doing and saying the ‘right’ thing, of not ‘pressuring’ the person to return to work or not, and concern about potential litigation. Concerns also related to other colleagues’ health and safety, particularly where machinery and equipment was involved, and around the possibility of having to terminate the employee’s contract:

That was the main concern; that we didn’t cause another accident in any way. We’ve got moving machinery, rollers running fabric that can pull a person into the machine…..nobody wants that now or wanted it at any stage in the process; we were all really concerned about having to face that eventuality (that the employee might not be considered safe to return). (Participant 7, line manager)

## Characteristics of Employees Returning to Work After Stroke

### Personal Characteristics

Participants referred to several personal qualities and characteristics of the employee with stroke which were associated with their return to work. Those important to the participants included that the employee had a good work ethic and a positive outlook, was hard-working and held a responsible job. Not being ‘too old’ was seen as an advantage although a long history of employment with the company was also viewed positively. Other characteristics seen as beneficial were that the employee liked their job, fitted in well with the team, were easy to work with, were enthusiastic and popular and had a good relationship with their manager.

But he’s a very enthusiastic employee and it’s been fairly simple actually because he’s been very enthusiastic and wanting to come back so it’s been fairly easy to make adjustments for him. (Participant 10, occupational health manager)

### How Employers Perceive the Effect of Stroke on the Individual Employee

Employers described a wide variety of symptoms which affected the individual employee’s ability to work including memory problems, confusion, speech and swallowing problems, fatigue and low stamina, numeracy, literacy, planning, vertigo, pain, concentration, emotional lability, change in character, communication and difficulty in handling conflict.

What she was telling me was that, like I’d have a communications meeting every week, she’d come to the communications meeting, she’d hear the first sentence, and then she wouldn’t hear any of it for the rest of the hour. (Participant 5, line manager)

Generic psychosocial barriers to work such as low self-esteem and confidence were more likely to be referred to by those who had no direct experience of supporting an employee with stroke return to work. Those participants who had active experience of an employee with stroke made more reference to the psychological and or cognitive effects on the individual’s work ability than any physical limitations. However there was also a view that employers would be more likely to consider the physical impacts of stroke, at least initially:

I suspect most of them would look at the physical stuff, the – you know, potential that the arm or the leg doesn’t work…… I doubt that they think about cognitive – and obviously I doubt they think about the fatigue and of course the other one is, is that people are often a bit more emotional and a bit easier to cry. (Participant 15, disability adviser)

### Motivational Factors

The individual’s personal motivation to return to work was considered a positive and necessary characteristic particularly where individuals hadn’t received support from outside the workplace, for example because clinicians may not have the time or ability to motivate patients to return to work, and the benefit system may be perceived as a disincentive. Again, individuals’ psychosocial barriers to work were more likely to be referred to by those who had no direct experience of supporting an employee return to work after stroke:

I wouldn’t necessarily call them disabilities, in that respect I would say there are actually normal aspects of being out of work and of being ill such as effects on confidence and self-esteem……. Again it gets back to - you cannot ignore the psycho-social factors. (Participant17, occupational health physician)

The participants who did have experience of supporting a stroke survivor were more likely to refer to the employee’s high drive and motivation. Motivation to return to work could be associated with the individual’s need to test their recovery and because it signified a return to normality. It might also be connected to financial insecurity (e.g. economic uncertainty, expiry of sick pay), perceived loss of status, feelings of guilt due to a belief that they were burdening their colleagues or associated with gender or cultural factors.

Obviously he wanted to do more, well because he’s …. I shouldn’t say this, this is a bit.… but he’s Scottish, he’s a man, he didn’t want to feel as if he was not capable anymore and he wanted to do more.

(Participant 13, line manager)

However this drive could lead to failure if the employee tried to return to work too soon (including returning to full hours and duties), or was unable or unwilling to ask for help.

### Awareness/Acceptance of Limitations

Closely associated with motivation to return to work was the ability of an employee to be aware of and/or accept limitations in work capacity following a stroke. Not being able to perform to their previous level of ability might result in the individual deciding not to return to work at all. An employee might not even have considered the option of a graded return or modified work and might perceive such arrangements as a sign of weakness. Sometimes employees had actually returned to work before they were able to appreciate the extent of their limitations, and realise they may have returned to work too soon:

And I think maybe coming back to work helped her realise that as well because I think that’s a bit more where she is now and her words to me was, I need to put this job behind me now and move on and do what I am capable of doing, rather than trying to do what I used to do. (Participant 5, line manager)

## Communication

### Asking for Help

Associated with the employee’s motivation and awareness/acceptance of limitations was the individual’s ability and/or willingness to ask for help at work. This may be due a pre-existing character trait or a need to ‘prove’ oneself and may result in the employee not completing tasks, making mistakes or appearing unwell. Managers sometimes found this difficult to address with the employee particularly as it was reliant on the individual sharing this information with them.

She’s just not honest about how she probably is feeling. But I can see it in her face. And in fact someone sometimes, the rest of the team will say gosh, she looks really tired today. And I’ll think yeah, you know, she does. And that’s when she needs to be saying. (Participant 3, line manager)

Where teams had worked together for some time, and were supportive, it might be seen as more acceptable to ask colleagues for help. Reticence to draw attention to limitations might also reflect an uncertain economic climate and the perception that those with a health condition such as stroke were at greater risk of redundancy.

### Consent and Confidentiality

Issues around consent and confidentiality could create barriers to successful return to work. Individuals might be unwilling for their manager to disclose certain information about their condition to their colleagues, or for occupational health to disclose information about their consultation to their manager. This could make it more difficult for managers and colleagues to fully understand and support the individual.

And the other thing that’s quite difficult is the other staff because if you’re managing the team and you have to make a load of adjustments for this person who’s coming back to work, as much as people can see ‘oh she’s had a stroke blah blah she’s got to come back’ the resentment always happens so you’ve got, ‘well why isn’t she taking the notes up to x floor, why am I?’ – and they [line managers] get a bit worried about that …but they can’t tell everybody why that decision’s been made because obviously that’s confidential to that person. (Participant 16, Human Resources manager)

Consent issues also led to delays where employers/occupational health were awaiting reports from GPs or consultants to inform decisions about the individual’s return to work plan, or if employers wanted to contact clinicians about their treatment progress. Participants described ‘short-circuiting’ these barriers by using the employee as a conduit of information.

Sometimes you can get the patient on your side and you can say, “Look, when you see your physio next, or whoever, can you ask them, can they put anything in writing?” and sometimes the physios will do that. (Participant 12, Occupational health nurse)

Consent and confidentiality also restricted the ease of communication between GPs and employers.

### Communication Between Healthcare and the Workplace

Associated with consent and confidentiality was the degree of communication between clinicians and employers. Delays in receiving reports from GPs and consultants were not always related to consent, but due to faulty systems. There were also costs involved in obtaining reports. In many cases communication was limited and attempts were usually driven by employers rather than clinicians.

This is no kind of criticism but I cannot actually remember a situation, in this type of arena, whereby a kind of therapeutic from a rehabilitative kind of position, has come to us you know, this is where we are, what can you do to help? I mean its seventy-six thousand employees we have.

(Participant 17, Occupational health physician)

Where two-way communication had been initiated by clinicians regarding the return to work of a stroke survivor, this was limited to those employees who had been recruited to the CLAHRC feasibility study.

Communication between occupational health and managers was likely to be one-way and through written report, particularly in very large organisations and where occupational health was out-sourced. Where occupational health was in-house, joint meetings between occupational health, HR, management and the employee were more likely to occur.

## Knowledge and Information

### Employers’ Understanding of Stroke

Although stroke is a common health condition, employers generally had limited experience of supporting individuals return to work after a stroke, even in large businesses. Occupational health departments may not record the number of current or previous employees with stroke. Employers may have had no previous experience of stroke, or their understanding may be informed by first, second or third hand experiences of family and friends, which may not be relevant to the person in question.

Twenty-seven years in production management and this is the first time I’ve really had to deal with such a severe medical case – disability as you say, so my knowledge is very lacking on it. (Participant 7, line manager)

There was an appreciation however that stroke affects individuals differently.

### How Employers Gain Knowledge About Stroke

Apart from knowledge gained directly from the stroke employee, the internet was likely to be the first place employers would look for information about the condition. Where in-house occupational health services were supported by a visiting GP they were more likely to be a reliable source of information, as the GP was more likely to have treated stroke patients as part of their regular practice. Occupational health on-line networks were cited as another useful way of sharing information and experiences. However, even where occupational health was provided, managers did not appear to use it as a primary source of information about health conditions such as stroke, particularly when the service was out-sourced as this line manager from a large organisation describes. The return to work had not been sustained:

I did a lot of research through the internet, it’s not hard now to find out exactly what dysphasia is and what the effect is. (Participant 5, line manager)

### Awareness of Disability Management

There was a lack of awareness of disability management and equality legislation among employers working in smaller businesses. Some had not considered this aspect at all, others were aware that their knowledge of their responsibilities was limited.

I don’t know what my legal requirements are, can I just – can I get rid of them? Can I just ask them to leave because it’s not working out? I don’t know. (Participant 1, managing director)

There was a fear of being accused of discrimination or being seen as ‘cashing in’ on disability by raising the topic with an employee. Whereas some employers considered that they treated each an employee as an individual with their own strengths and weaknesses, other employers felt uncomfortable about managing individuals ‘differently’. Perceptions of the how the employee might perceive being ‘disabled’ was also discussed in that the perceived stigma attached to the term ‘disability’ might prevent the individual from seeking support from services for ‘disabled’ people.

### Awareness of Existing Support Services

Employers from smaller businesses were also less aware of how to access support from existing avenues such as through statutory employment services, or local health service initiatives. Larger organisations were more likely to have experience of involving these services. Three participants had experience of external support services that had not liaised effectively with the employer and/or had suggested adjustments that were inappropriate. Participants were unclear about the responsibilities and roles of the personnel involved. The systems were perceived as difficult to access.

I know they’re there, but I wouldn’t know how to even start looking for them and that sort of thing. It just seems like too much hard work for me to sort it out…. there’s too much red tape and it’s just easier for us to actually sort something else out. (Participant 10, occupational health manager)

Only one employer had contacted his employee’s hospital occupational therapist to advise him on return to work, as a result of his own prior experience of rehabilitation services.

## Experiences and Perceptions of Other Stakeholders

### GPs/Physicians

GPs and physicians were not perceived as having a major role in supporting their patients’ return to work after stroke other than in issuing ‘fit notes’ (the recently revised sickness certificate which GPs can use to advise employers on an individual’s fitness to work) and sending reports to employers when requested to (for which there is a charge). One participant, a stroke survivor who ran his own small business, described himself as being ‘lucky’ in having a supportive GP. However, it was perceived as easier to obtain a report from a GP than from a hospital physician. Communication between employers and GPs was rare. An occupational health manager reported that it was fortuitous that the company doctor was also the GP of the employee returning to work following stroke, as this meant that they could more easily obtain the information they wanted from the medical records. It was perceived that GPs might be too ‘busy’ to address work issues, that it was ‘easier’ for GPs to sign patients off as ‘not fit’ to work, and that GPs could do more to encourage patients to contact their occupational health department. Fit notes had the potential to be helpful as they could offer greater reassurance to insurance companies and for employers to feel ‘covered’. However, others considered that fit notes had made little difference to practice. There was little reference to the use of the fit note in advising employers on how to manage employees with stroke.

### Occupational Health (OH)

Medium and small-sized businesses were unlikely to have access to occupational health support for employees returning to work after stroke. Where they were available, perceptions of occupational health services varied, but they were generally considered valuable. However, the associated costs meant that it might be used discreetly. In most cases the assessment of the employee’s capacity to work was made by talking to the employee and combining this with reports from GPs and physicians where available.

I don’t think they [occupational health] tend to examine them as such themselves. And a lot of it is done by enquiry of the GP and/or the consultant. So a report will go off to the GP, you know, in order to answer some of the queries so that - I mean obviously we would always take what our occupational health unit say to us in terms of their fitness or otherwise for work. But obviously they need to go to GPs and consultants to gauge the level of ability that person’s got. (Participant 4, Human Resources manager)

Worksite visits to assess the individual job, either with or without the employee, were not standard procedure and more likely when OH services were ‘in-house’. In very large organisations where OH was contracted out, the majority of OH assessments were undertaken by telephone, and were restricted to a set duration. Several different personnel might be involved at different stages of the assessment process, and there was little opportunity for discussion. One manager felt that they did not have sufficient information about the effect of the stroke on the employee from the occupational health reports.

### Management (Human Resources and Line Managers/Supervisors)

Participants from businesses that did not have access to a formal Human Resources (HR) department felt that they were at a disadvantage as they considered it a source of specialist information and support. However, even where there was a Human Resources department, line managers were more likely to take on the main responsibility for supporting their employee return to work after stroke. This might be due to low levels of staffing in Human Resources, but also because stroke was seen to be a ‘genuine’ condition rather than in cases where there was some doubt over the legitimacy of sickness absence:

I think the ones HR are looking for are the ones that maybe they think ‘they’re spinning this one out a bit’. (Participant 3, line manager)

Whether or not an employee was referred to occupational health was generally the line manager’s decision rather than HR. For some cases referral might be ‘triggered’ automatically through sickness records so that people did not ‘slip through the net’, but where employees were at work but struggling, referral tended to rest with the manager even if self-referral was available. The importance of good supervision and support processes was recognised as line managers may not always have the skills or attitudes to identify and manage health-related problems, or refer to HR for support.

A lot of the time – and this is always the issue – it’s about the competence of the line manager…….I mean, myself and HR, we are still advisers to the business or to the organisation, so it is ultimately the line manager’s responsibility for making sure all the steps are followed and the contacts kept. (Participant 12, occupational health nurse)

Where RTW might be a lengthy staged process, it was anticipated that HR would want to be provided with a clear strategy of RTW and kept informed of progress.

## Integrating Healthcare in Return to Work

### Content and Delivery

Four participants had experienced the involvement of healthcare professionals from outside the workplace (in this study occupational therapists) in supporting an employee with stroke return to work, in three cases through the CLAHRC feasibility randomised controlled trial described earlier and in one case through a company insurance scheme. Those who did not have this experience were not necessarily able to conceptualise what this intervention might consist of. Where participants had newly employed an individual following their stroke, they perceived a service that could be accessed on an ad hoc basis would be useful, perhaps linked to existing employer organisations such as Chambers of Commerce, with different levels of support such as an information website, telephone helpline or someone to visit the workplace. Larger organisations supported the principle of an NHS-led RTW intervention but were unsure how it would fit with existing occupational health providers who might ‘think that they’re being replaced’. There was concern that roles and responsibilities were made clear, and that unrealistic expectations weren’t raised with the individual employee about modifications that might be made. There was a perceived risk that different professions might each consider that ‘they know best’ and overlook the need for partnership working, implying an element of competition in the return to work stakes. The possibility that an NHS-led intervention might be able to liaise more effectively with GPs and other clinicians was seen as an advantage.

Having your own occupational health advisor working or trying to work with the NHS is near on impossible. It just doesn’t work. (Participant 8, managing director)

Participants who had experience of occupational therapists supporting an employee felt that they had benefited from advice and information on how stroke affected the individual, support regarding work modifications including phased returns and worksite visits, and in supporting both the employee and the manager:

She actually came out and visited the premises and walked the production line with me, looking at what may cause him a problem. I’m not sure how important it was for [the employee’s] overall rehabilitation but regarding my understanding of how to judge what [the employee] was doing was massive. (Participant 7, line manager)

There was a perception that VR therapists had a detailed understanding of the condition and symptoms, helped the employee to set realistic goals and could act as a mediator. The ideal duration of such an intervention was unclear. It was appreciated there was a balance to be met between providing sufficient support given that problems could arise sometime after an individual had returned to work, and the need for the manager and employee to assume responsibility.

### Who Might Pay?

There were mixed opinions as to who should pay for a healthcare-led RTW service. Some considered that it should be provided by the NHS or directly from the government.

I think it has to be the government, it has to come from outside simply because they want to get people back, re-employment, what do they call it, people that have got a disability and stuff and getting them back to work. Yes it’s alright for your big companies, but the smaller companies obviously would like to get these people in, obviously can’t afford it. (Participant 2, line manager)

One participant felt that the employee should receive funding directly to buy in the RTW support they needed in order to give the individual more autonomy. Smaller businesses were less likely to think they themselves should pay, although one, when asked whether the RTW intervention they had received was worth £500, agreed that it might be cost-effective. One employer who had already experienced paying privately for an occupational health-sourced intervention reported that he would be willing to pay towards an NHS-led intervention instead if he thought it might lead to better communication with the GP and hospital care. Larger businesses and organisations already funding occupational health services considered it unlikely that they would pay for any additional healthcare-led services.

## Discussion

This study has demonstrated the complex emotional and practical issues faced by employers when supporting an employee return to work after stroke and the range and quality of support networks that they access. The findings have also informed the thinking as to whether and how a therapy-led VR service for stroke patients could be delivered and its potential impact.

### The Needs of the Employer

Employers, and particularly line managers, are pivotal to the return to, and retention of, work after stroke. Our findings suggest that it is a role for which most are unprepared. Although every year in the UK more than 30,000 people of working age experience a stroke it was perceived by the participants in this study as an unusual, sudden and unexpected event. This finding supports those reported by Culler et al. [25] that most employers’ experience of stroke occurs when employees who have had a stroke return to work. Employers therefore have little prior experience to guide them and as stroke affects individuals differently, any previous experience is not necessarily transferable. Despite this responsibility, many employers, particularly those in smaller businesses lacked awareness in obtaining relevant information about stroke, support services and disability management in the workplace. Even where occupational health services were provided, understanding of the condition and how it affected the individual was often guided by what the stroke survivor says, and the advice of the GP, which may not be accurate or objective. GPs often feel ill-equipped to advise employers on patients’ work ability and are constrained by their advocacy role [27]. Black and Frost [18] report that in longer-term and complex sickness cases, employers say they need ‘independent, bespoke advice’ but that most GPs do not consider themselves expert in this area.

Also, as our findings indicate, employees with stroke may be unable or unwilling to identify their needs and limitations or struggle to communicate them effectively, for a range of possible reasons, which are reported in other studies. These include speech problems, lack of insight or a high motivation to return to/remain at work for reasons of, for example, to benchmark their recovery [22], guilt at burdening colleagues [5], financial insecurity and fear of losing their job [28]. Decisions made concerning risk assessment, work accommodations such as altered hours and duties, and monitoring of performance may therefore be inappropriate or inadequately applied. There are few studies of the employers’ perspective in RTW after stroke, but supervisor’s views of the importance of knowledge and information about the employee’s condition have been reported in studies of RTW following depression [29] common mental disorders [30] and other chronic conditions [31]. Not all employers have access to support from Human Resources. Where Human Resources departments are provided they are more likely to be involved in cases of complex health conditions, however this is not necessarily the case; according to Haafkens et al. [31] many of the personnel tasks previously performed by HR managers have been devolved to the line manager. There is limited guidance and training on this role available to managers [31], and Cunningham et al. [32] report that line managers tend to take on the responsibility for disability management without asking for support from HR or OH services. However, as reported by Holmgren and Ivanoff [23], although supervisors may want to be supportive, they may struggle to balance work demands and limited resources whilst finding suitable work tasks for employees with health conditions and disabilities.

Other studies have reported on the ‘burden’ experienced by employers in RTW following illness such as cancer [33]. One of the unexpected findings in this study was that employers could instead perceive the RTW process as a positive and rewarding experience. The benefits of ‘caregiving’ have been recognised in relation to families of individuals with for example dementia [34] and cancer [35] but there is little known about the benefits of supporting an employee with a health problem or disability, and how these might be promoted. As this study has shown, providing this support can be a stressful experience for those involved, and the need to promote the health and well-being of both the employee and employer has been recognised [36]. As reported by Holmgren and Ivanoff [23] ‘when rehabilitation succeeds, it is not only the sick-listed employee and the supervisor that come out strengthened, but the whole working team’. However, in this study, it was equally important for employers to feel satisfied that they had covered every avenue, even when return to work was not possible. Exploring the use of conceptual models of caregiving such as that presented by Carbonneau et al. [37] from the perspective of employers involved in RTW and work retention may be an area of future research.

### The Potential for Partnership Working Between Health Services and Employers

Given that many employers have limited understanding of and expertise in managing the problems experienced by employees following stroke, the lack of even one-way communication between employers and clinicians, let alone two-way dialogue or discussion, and the lack of support provided by health service providers as found in this study is remarkable. Apart from those participants who had been involved in the feasibility randomised controlled trial, clinicians had rarely initiated contact with patients’ employers. The reason for this needs further exploration, particularly as work-focused healthcare is a now a government priority [38]. Attempts by employers to contact clinicians were generally impeded by policies and procedures rather than encouraged. Communication was usually unidirectional and via the employee. Previous studies have reported on the need for better communication between, for example, GPs, physicians and occupational health [39, 40] and supervisors [30]. In a retrospective French study of stroke [41], close co-operation between occupational health services and patients’ rehabilitation team was a prognostic factor in return to work, and in a qualitative study by Culler et al. [25] employers had found interaction with VR staff helpful in hiring staff. Studies to identify how communication and dialogue can be facilitated, and what the most effective mechanisms are, are urgently required. As reported in a recent review of sickness absence in the UK, ‘there are no strict rules about health services focusing on RTW or communicating with employers’ [18]. It is also important to note that only between 12 % and 34 % of the workforce is estimated as being covered by occupational health arrangements [42]. A free telephone help-line for businesses with fewer than 250 employees has been piloted however the long-term provision of this service is unclear [43].

Where employers had experienced support from clinicians regarding their employee’s return to work, particularly through worksite visits, this was generally viewed very positively. However, while some employers would be willing to pay for such a service, others were not. Some considered that the role could be easily integrated within their existing systems of disability management whereas others were concerned that it might lead to conflict. Other studies have suggested [38] that in order to improve partnership working between employers and rehabilitation professionals, a consensus is required as to professional roles and responsibilities. Integrated approaches have shown to be effective for example in the return to work of employees with back pain [44], however, the majority of studies have been conducted where the costs of sickness and work disability are closely connected to the employer. In North America the Supported Employment model (where job ‘coaches’ are integrated with the healthcare team, employer and employee) developed for people with learning disabilities [45] and evaluated successfully in mental health [46] is well-developed and has shown promise in traumatic brain injury TBI [47] but has yet to be evaluated as part of an RCT [48, 49] and has not been implemented or evaluated in the UK following stroke. Research is needed to test integrated approaches with patients whose health condition is not work-related and in countries where employees are not covered by job protection legislation and insurance.

### Application of the Biopsychosocial Model

Finally, it is known that physical, emotional, cognitive and psychological problems may result from stroke and affect work activities [10]. In our study, physical limitations were less frequently referred to. This may be a feature of our sample in that those interviewed had less experience of people with greater physical impairment or because other problems had greater salience at work and were perceived as more complex to manage. Participants did describe for example how speech, motor and sensory problems had affected some employees, however, details of the specific deficits and the extent to which they were experienced by each stroke survivor were not collected as part of the study and could in hindsight have aided understanding. It may be that those who had no experience of engaging with for example government employment services or health-based VR and who had managed their own return were less likely to present with physical problems. Also of interest was that more generic ‘psychosocial’ factors such as self-esteem and job satisfaction were referred to as barriers to return to work. There has been considerable evidence published in recent years regarding psychosocial barriers to return to work, particularly with regard to mild-moderate mental health and musculoskeletal problems [7]. Although these are important factors, it is essential that the ‘bio’ elements of the biopsychosocial model are given equal primacy. Likewise, as reported by Soklaridis et al. [50] care should be taken not to ‘psychosocialize’ workers when these problems may arise from system and communication barriers rather than the individual, or to medicalise a natural reaction to returning to work after a period of absence.

### Strengths and Limitations of the Study

This study has added important findings to the current evidence base for employers’ perspectives in return-to-work following stroke. Although in four cases the data collected was hypothetical rather than stroke-specific, the remaining fourteen participants all had direct experience of supporting a stroke survivor returning to work. Culler et al. [25] reported on the difficulty in recruiting employers to research studies of RTW after stroke. Their study interviewed seven employers all of whom had limited specific experience of stroke, and were from medium to large enterprises only. Although this study recruited a convenience sample, and data saturation may not have been reached, the range of stakeholders interviewed has provided diversity. There is very little research concerning return to work following stroke, and even less concerning the employer perspective. Lock et al. [21] reported that they did include employer interviews in their study of work after stroke but their findings have not been published. In this study we experienced difficulty both in accessing employers and in recruiting them once identified. This may in part be due to the fact that although stroke is a common health condition, it is unlikely that an individual manager will experience this condition in their team on a regular basis in contrast with conditions such as depression or musculoskeletal disorders. However, issues of confidentiality and ethical constraints were also barriers to recruitment. Employers may be reluctant to participate if they perceive that their management of return-to-work will be under scrutiny, or may impact on their relationship with the employee. These factors need to be addressed in future studies. A further limitation is that the study was conducted in a country with a national healthcare system but where most employees are unable to access support from occupational medicine or vocational rehabilitation practitioners. The findings may therefore be less generalizable to other settings.

## Conclusion

Employers face complex emotional and practical issues when helping an employee return to work after stroke, for which many lack knowledge and experience. A number of factors can facilitate or hinder the employer in this role, including the motives underlying the employee’s decision to return, the relationship between the employer and employee, the functional effects of the stroke in relation to work tasks and the ability and willingness of the employee to ask for help. The range and quality of support networks that employers can access is variable. Many receive no support at all while some are able to access support from within the workplace, but few are able to obtain stroke-specific expertise to guide them. Advice and support from clinicians specific to the individual with stroke is welcomed by employers, but there are questions as to how such support could be funded and integrated within existing service provision which further studies need to address. There appears to be a complex array of relationships that ideally should align to orchestrate a successful return to work following stroke. However, participants who had received support from a health care professional with knowledge of both VR and stroke appeared to benefit. How these relationships can be fostered and services co-ordinated and financially sustained requires urgent attention.
